# Preclinical development of a microRNA-based therapy for intervertebral disc degeneration

**DOI:** 10.1038/s41467-018-07360-1

**Published:** 2018-11-28

**Authors:** Ming-liang Ji, Hua Jiang, Xue-jun Zhang, Pei-liang Shi, Chao Li, Hao Wu, Xiao-tao Wu, Yun-tao Wang, Chen Wang, Jun Lu

**Affiliations:** 1 Department of Orthopaedic Surgery, Zhongda Hospital, School of Medicine, Southeast University, 210009 Nanjing, China; 2 Department of Spine Surgery, The First Affiliated Hospital of Guangxi Medical University, Nanning, 530000 China; 3 Key Laboratory of Model Animal for Disease Study of Ministry of Education, Model Animal Research Center, Collaborative Innovation Center of Genetics and Development, Nanjing University, Nanjing, 210093 China

## Abstract

Understanding the molecular mechanisms regulating the maintenance and destruction of intervertebral disc may lead to the development of new therapies for intervertebral disc degeneration (IDD). Here we present evidence from miRNA microarray analyses of clinical data sets along with in vitro and in vivo experiments that miR-141 is a key regulator of IDD. Gain- and loss-of-function studies show that miR-141 drives IDD by inducing nucleus pulposus (NP) apoptosis. Furthermore, miR-141 KO in mice attenuated spontaneous and surgically induced IDD. Mechanistically, miR-141 promotes IDD development by targeting and depleting SIRT1, a negative regulator of NF-κB pathway. Therapeutically, upregulation or downregulation of miR-141 by nanoparticle delivery in IDD model aggravated or alleviated experimental IDD, respectively. Our findings reveal a novel mechanism by which miR-141, in part, promotes IDD progression by interacting with SIRT1/NF-κB pathway. Blockade of miR-141 in vivo may serve as a potential therapeutic approach in the treatment of IDD.

## Abstract (web summary)

Intervertebral disk degeneration (IDD) is characterized by changes in the nucleus pulposus (NP) extra cellular matrix that compromise disk structural integrity. In a miRNA screen of human IDD patient NP tissue, the authors identify deregulated miR-141 and show that direct injection of nanoparticle-coupled miR-141 into the NP alleviates IDD in mice.

## Introduction

Low back pain (LBP) is a leading cause of disability worldwide and imposes an enormous clinical and socioeconomic burden on society^1^. Although numerous potential causes are recognized, the disorder is strongly associated with intervertebral disc degeneration (IDD), which accounts for approximately 40% of all LBP cases^2^. The intervertebral disc (IVD), which lies between the adjacent vertebral bodies and provides load support, flexibility, energy storage, and dissipation in the spine, are composed of a gel-like nucleus pulposus (NP) surrounded circumferentially by a fibrocartilagenous annulus fibrosus (AF)^3^. The NP plays critical roles in maintaining homeostasis by secreting a complex extracellular matrix (ECM) consisting predominantly of type II collagen and proteoglycans, which are indispensable to the physiological viscoelastic properties of the IVD^4^. IDD originates in the NP and is characterized by ECM component alterations. During the process of IDD, the most dramatic cellular and biochemical change is that the centrally located NP cells undergo phenotypic transition and are replaced by smaller fibrochondrocyte-like cells, resulting in decreased proteoglycan synthesis, and an overall shift towards synthesis of a fibrotic matrix and events that compromise the structural integrity of discs^5,6^. Although the precise pathogenesis of IDD remains elusive, it is generally thought to be the result of microenvironmental changes within the IVD induced by multiple factors, including genetics, aging, sex, a predisposing injury, and environment^7,8^. The major pathological hallmark of IVD degeneration is increased production of degradative enzymes coupled with decreased synthesis of ECM resulting from a homeostatic imbalance between anabolism and catabolism^9^. Therefore, an enhanced understanding the molecular mechanisms underlying this imbalance has the potential to identify new therapeutic targets for IDD.

There is increasing evidence supporting the role of microRNAs (miRNAs) in the processes that leads to IDD^10–12^. miRNAs are a class of non-coding RNA molecules that play a central part in cell differentiation, proliferation, and survival by binding to complementary target mRNAs, resulting in mRNA translational inhibition or degradation^13^. During development, miRNA expression is tissue-specific, which suggests that miRNAs may help to specify and maintain tissue identity. Thus establishment of a miRNA expression profile is important for investigating the underlying functional mechanisms for IDD, and a better knowledge of their expression patterns could reveal molecular signatures that can be developed as therapeutic targets as well. Of note, in the short time since the discovery of miRNAs, therapeutic approaches to manipulate them have progressed from bench to bedside, with some successful phase I trials and ongoing phase II/III trials^14,15^. In this study, a miRNA microarray strategy was applied to identify differentially expressed miRNAs in NP tissues by comparing miRNA profiles between IDD patients and normal controls. miR-141 is shown to be most significantly upregulated. Subsequent in vitro and in vivo studies demonstrated that miR-141 plays a key role in the pathogenesis of IDD. Mechanistically, the crosstalk between miR-141 and SIRT1/NF-κB pathway is a key determinant of IDD. Of crucial importance, local delivery of nanoparticles (NPs) carrying miR-141 inhibitor alleviates IDD. Clinically, miR-141 level are associated with grade of disc degeneration. These data collectively indicate that miR-141 could be a target for therapeutic intervention against IDD.

## Results

### Discovery of IDD-associated miRNAs by microarray

**Fig. 1:**
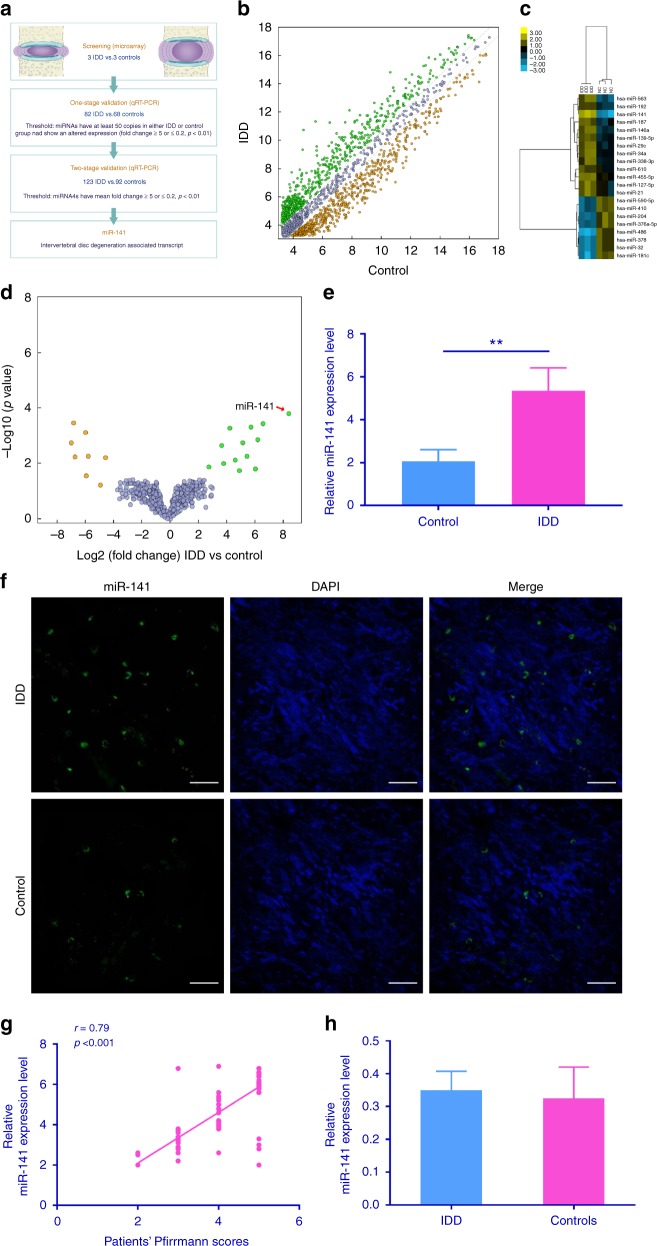
Identification of differentially expressed miRNAs in NP tissues from IDD patients. **a** Selection strategy of miRNAs in degenerative NP tissues derived from microarray-based profiling. **b** Scatter plot of miRNA expression profile between IDD patients and controls (green dots, upregulation more than two-fold; yellow dots, downregulation more than two-fold). **c** Heat map depicting 21 differentially expressed miRNAs (fold change >5 or <0.2, Benjamini–Hochberg-corrected *p*). **d** Volcano plot illustrating the biological and statistical significance of differential miRNA expression levels between IDD patients and controls. The negative Log10-adjusted *P* values (*y* axis) are plotted against the Log2 fold changes in expression (*x* axis). Green dots indicate the upregulated (right side) and yellow dots indicate downregulated (left side) miRNAs. miR-141 is indicated. **e** Compared with controls (*n* = 163), miR-141 expression level was upregulated in IDD patients (*n* = 208). **p < 0.01 by Mann–Whitney *U* test. **f** FISH analysis of NP tissues from IDD patients demonstrated increased level of miR-141. Scale bar = 25 μm. **g** The miR-141 expression level in NP tissues from IDD patients was positively correlated with the disc degeneration grade (*n* = 208; *r* = 0.79, *p* < 0.001). **h** Concerning miR-141 expression profile in bone surrounding disc, no significant difference was observed between IDD and controls. NP nucleus pulposus, IDD intervertebral disc degeneration, FISH fluorescence in situ hybridization. Data shown as mean and error bar represents s.e.m.

To decipher the roles of miRNA in IDD, we first examined the miRNA expression profiles with miRNA microarray on three NP tissues from IDD patients vs. three normal NP tissues from fresh traumatic lumbar fracture patients (Fig. 1a). The miRNAs were detected by microarray (Fig. 1b). Unsupervised clustering analysis with these significantly dysregulated miRNAs was able to distinguish IDD patients from controls (Fig. 1c, d). We first tested these candidate miRNAs using an independent cohort of 82 IDD patients and 68 controls. Only miRNAs with a mean fold change >5 or <0.2 and a *p* value <0.01 were selected for further analysis. Using the above-mentioned criteria, miR-146a, miR-141, miR-21, and miR-378 were observed to be significantly dysregulated (Supplementary Table 1). These four miRNAs were further evaluated by quantitative reverse transcriptase–PCR (qRT-PCR) using additional independent cohort comprising of 123 IDD patients and 92 controls. Of the four miRNAs, miR-141 was found to be significantly upregulated in IDD patients compared with controls (Supplementary Table 1). We therefore selected miR-141 for further investigation. qRT-PCR results demonstrated that miR-141 expression level was upregulated in 208 NP tissues from IDD patients compared with 163 controls (Fig. 1e), which was further confirmed by fluorescence in situ hybridization (FISH; Fig. 1f). Moreover, the expression of miR-141 in NP tissues from IDD patients was correlated with the disc degeneration grade (*n* = 208; *r* = 0.79, *p* < 0.001; Fig. 1g). Using quantitative PCR (QPCR), miR-141 expression level was analyzed in the surrounding bone from IDD patients and controls. The result showed that no significant difference was observed between IDD and controls with respect to miR-141 level in the surrounding bone (Fig. 1h). These findings suggest the possibility that miR-141 have disease-specific effects in IDD.

### MiR-141 knockout (KO) in mice inhibits spontaneous and surgically induced IDD progression

**Fig. 2:**
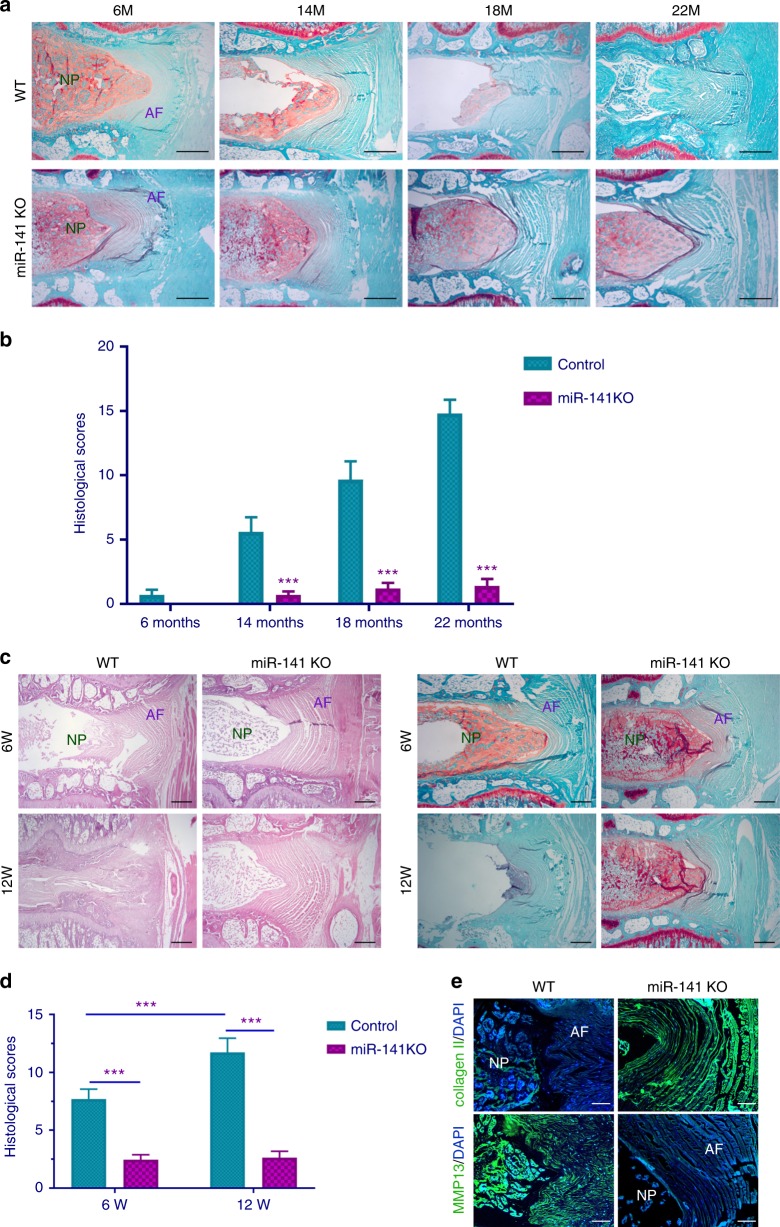
Genetic deletion of miR-141 suppresses spontaneous and surgically induced IDD. **a** Safranin O staining of intervertebral disc from 6-month-old mice (WT, *n* = 10; miR-141 KO, *n* = 10), 14-month-old mice (WT, *n* = 10; miR-141 KO, *n* = 10), 18-month-old mice (WT, *n* = 10; miR-141 KO, *n* = 10), and 22-month-old mice (WT, *n* = 10; miR-141 KO, *n* = 10). Scale bar = 50 μm. **b** The histological grades evaluated at 6, 14, 18, and 22 months in WT and miR-141 KO mice. ***p < 0.001 by unpaired two-sample Student’s *t* test. **c** HE and Safranin O staining of disc sections in WT and miR-141 KO mice obtained at 6 and 12 weeks after surgery (WT, *n* = 10; miR-141 KO, *n* = 10). HE, scale bar = 50 μm; Safranin O, scale bar = 50 μm. **d** Histological score of WT and miR-141 KO mice, as measured through Safranin O result. ***p < 0.001 unpaired two-sample Student’s *t* test. **e** Immunostaining for collagen II and MMP13 in discs of WT and miR-141 KO mice. Scale bar = 100 μm. Data shown as mean and error bar represents s.e.m.

At the age of 6 months, miR-141 KO and wild-type (WT) mice had well-organized NP and AF (Fig. 2a). NP cells were mostly stellar shaped and exhibited normal staining. NP cells clusters were centralized with a layer of proteoglycan-rich matrix located at the periphery. The AF consisted of well-organized collagen lamellae and fibroblastic cells, which was localized in orderly stratified curves around NP. The endplates were continuous. At 14, 18, and 22 months of age, the WT mice showed disordered morphology of NP and AF. In contrast, degenerative changes were less severe in the age-matched miR-141 KO mice (Fig. 2a). The histologic scores were markedly decreased in miR-141 KO mice compared with that of WT mice (Fig. 2b), indicating alleviated destruction of IVD structure in miR-141 KO mice. These results indicate that miR-141 is a significant contributor of aging-associated spontaneous IDD initiation and progression.

To further elucidate the role of miR-141 in the development of IDD, surgically induced IDD model was established. No acute inflammation (interleukin (IL)-1β, tumor necrosis factor (TNF)-α, and IL-6) was detected in NP tissues from the 29, 31, and 32 G needle groups at 1 and 3 days after needle puncture. In comparison, IL-1β, TNF-α, and IL-6 expression levels were high in the 18, 21, and 23 G needle groups. Low IL-1β, TNF-α, and IL-6 expression levels were observed in 28-G needle group (Supplementary Fig. 1). As expected, histological analysis at 6 weeks post-surgery, miR-141 KO mice showed minimal degenerative changes in IVDs (Fig. 2c). However, control mice showed greater destruction in some regions of IVDs (Fig. 2c). This phenotype became more profound at 12 weeks post-surgery, where control mice, in comparison to miR-141 KO mice, showed significant and severe destruction of IVDs (Fig. 2c). These results were confirmed by the significant increase in the histologic scores (Fig. 2d). Consistent with this finding, immunofluorescent staining demonstrated that the expression of matrix metalloproteinase 13 (MMP13) was significantly reduced in the miR-141 KO mice (increased level of collagen II) compared with those in control mice at 12 weeks post-surgery (Fig. 2e). These results demonstrate that miR-141 deficiency in mice protects against surgically induced IDD.

### The effect of miR-141 overexpression or silencing on NP cell phenotype

**Fig. 3:**
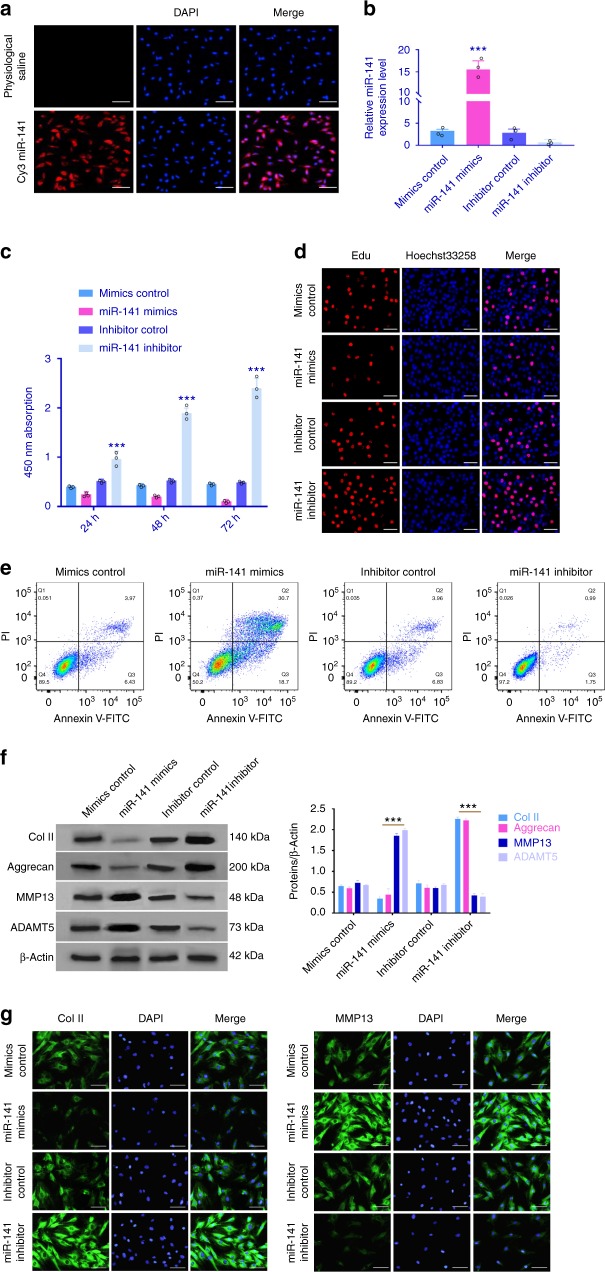
In vitro study of miR-141. **a** miR-141 transfecting cultured primary human NP cells as confirmed by Cy3. Scale bar = 100 μm. **b** Forty-eight hours after transfection of miR-141 mimics or inhibitor and their negative control, the cells were used for the following experiments. Transfection efficiency of miR-141 was analyzed using qRT-PCR. *n* = 3 replicates per group, ***p < 0.001 by one-way ANOVA test followed by Tukey’s post hoc. **c**, **d** Cell proliferation was analyzed in miR-141 mimics or inhibitor transfected cultured primary human NP cells using CCK8 and EdU assays. *n* = 3 replicates per group, ***p < 0.001 by one-way ANOVA test followed by Tukey’s post hoc. Scale bar = 100 μm. **e** Analysis of NP cells apoptosis was assayed by FCM. *n* = 3 replicates per group. **f** The expression levels of Col II, aggrecan, MMP13, and ADAMT5 were detected by western blot. Quantitative analysis was shown on the right, and three independent repeats were performed in each experiment. ***p < 0.001 by one-way ANOVA test followed by Tukey’s post hoc. **g** The representative Col II and MMP13 were detected by the immunofluorescence. Scale bar = 25 μm. Data shown as mean and error bar represents s.e.m.

To better understand the functional role of miR-141 in the pathogenesis of IDD, we transiently transfected miR-141 mimics or inhibitor into cultured primary human NP cells. Transfection efficiency was detected by Cy3-labeled miRNA (Fig. 3a), which was further confirmed by qRT-PCR (Fig. 3b). Our analysis indicated that miR-141 overexpression dominantly decreased cell proliferation compared with its corresponding miR-control at 24, 48, and 72 h time points, whereas miR-141 inhibitor markedly increased cell proliferation (Fig. 3c). 5-Ethynyl-2′-deoxyuridine (EdU) assay was performed to further explore the functional role of miR-141 in primary human NP cell proliferation (Fig. 3d). With respect to NP cell apoptosis, miR-141 inhibitor transfection was found to significantly decrease the amounts of NP cell apoptosis (Fig. 3e and Supplementary Fig. 3).

Next, using gain-of-function and loss-of-function studies, we further analyzed the effect of miR-141 expression on anabolic/catabolic markers. The expression of COL2A1 (Col II) and ACAN (Aggrecan) was increased in cultured primary human NP cells that were transfected with miR-141 inhibitor. In contrast, overexpression of miR-141 strongly increased ADAMTS-5 and MMP13 levels, whereas inhibition of miR-141 decreased ADAMTS-5 and MMP13 levels (Fig. 3f and Supplementary Fig. 2). This effect was further confirmed by immunofluorescence (Fig. 3g and Supplementary Fig. 4). Collectively, the data indicate that the downregulation of miR-141 promotes NP cell matrix synthesis and proliferation.

### Identification of SIRT1 as a target gene for miR-141

**Fig. 4:**
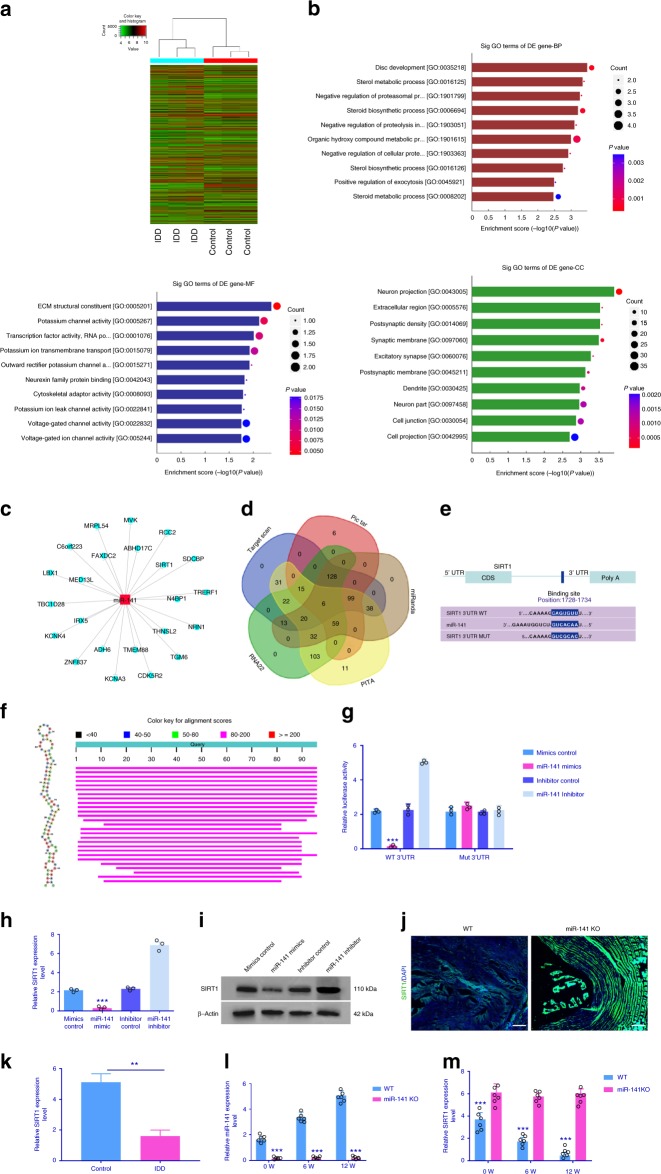
Identification of SIRT1 as a target of miR-141. **a** Microarray analysis showing genes that were differentially expressed between IDD and control. **b** Downregulated GO terms with the most significant *p* values for biological processes, molecular function, and cellular component. **c** Cytoscape was employed to confirm the target of miR-141. **d** Venn diagram displaying miR-141 computationally predicted to target SIRT1 by different algorithms. **e** Sequence alignment of a putative miR-141-binding site within the 3’UTR of SIRT1 mRNA shows a high level of sequence conservation and complementarity with miR-141. **f** High conservation of miR-141. **g** The wild- or mutant-type SIRT1 3’UTR reporter plasmid was co-transfected with miR-141 mimics or inhibitor into cultured primary human NP cells. Forty-eight hours after transfection, luciferase activity was measured. *n* = 3 replicates per group, ***p < 0.001 by one-way ANOVA test followed by Tukey’s post hoc. **h**–**j** SIRT1 expression level was detected by qRT-PCR, western blot in primary human NP cells, and immunostaining. ***p < 0.001 by one-way ANOVA test followed by Tukey’s post hoc. Scale bar = 100 μm. **k** Compared with controls, SIRT1 expression level in IDD patients was lower. **p < 0.01 by Mann–Whitney *U* test. **l**, **m** MiR-141 level in NP tissues from miR-141 KO mice was lower than that in WT mice, while SIRT1 level in NP tissues from miR-141 KO mice was higher than that in WT mice. **p < 0.01 by Mann–Whitney *U* test. Data shown as mean and error bar represents s.e.m.

Dysregulated mRNAs were also identified in IDD (Fig. 4a). All these genes were subjected to gene ontology (GO) analysis. Downregulated gene GO terms with the most significant *p* values for biological processes, molecular function, and cellular component were related to disc development (GO:0035218), ECM structural constituent (GO:0005201), and extracellular region (GO:0005576) (Fig. 4b). Moreover, miRNA–mRNA network using the Cytoscape software was constructed (Fig. 4c). By searching the potential targets of miR-141, we compiled all the predicted genes for Venn analysis (Fig. 4d). Based on above-mentioned results, SIRT1 was identified as the target of miR-141 (Fig. 4e and Supplementary Fig. 5). Additionally, miR-141 has a high level of conservation among species (Fig. 4f). To further confirm the functional interaction between miR-141 and SIRT1, we performed luciferase reporter assay analysis. Co-transfected SIRT1 WT (WT) with 141 mimics in cultured primary human NP cells was significantly lower than relative luciferase reporter activity of cells transfected SIRT1-mut (mutant) with miR-141 mimics (Fig. 4g). This effect was further supported by gene expression and protein expression in cultured primary human NP cells and mouse disc tissues (Fig. 4h–j). SIRT1 expression level was also detected by QPCR in NP tissues from 208 IDD patients and 163 controls, and the result demonstrate that SIRT1 expression level in IDD patients was lower than that in controls (Fig. 4k). At 0, 6, and 12 weeks, the levels of miR-141 and SIRT1 were investigated in NP tissues from WT and miR-141 KO mice, and the results show that miR-141 level in miR-141 KO mice was lower than that in WT mice, while SIRT1 level in miR-141 KO mice was higher than that in WT mice (Fig. 4l, m). These results validate SIRT1 as a direct target of miR-141.

### MiR-141 regulates IDD by modulating SIRT1/nuclear factor (NF)-κB signaling pathway

**Fig. 5:**
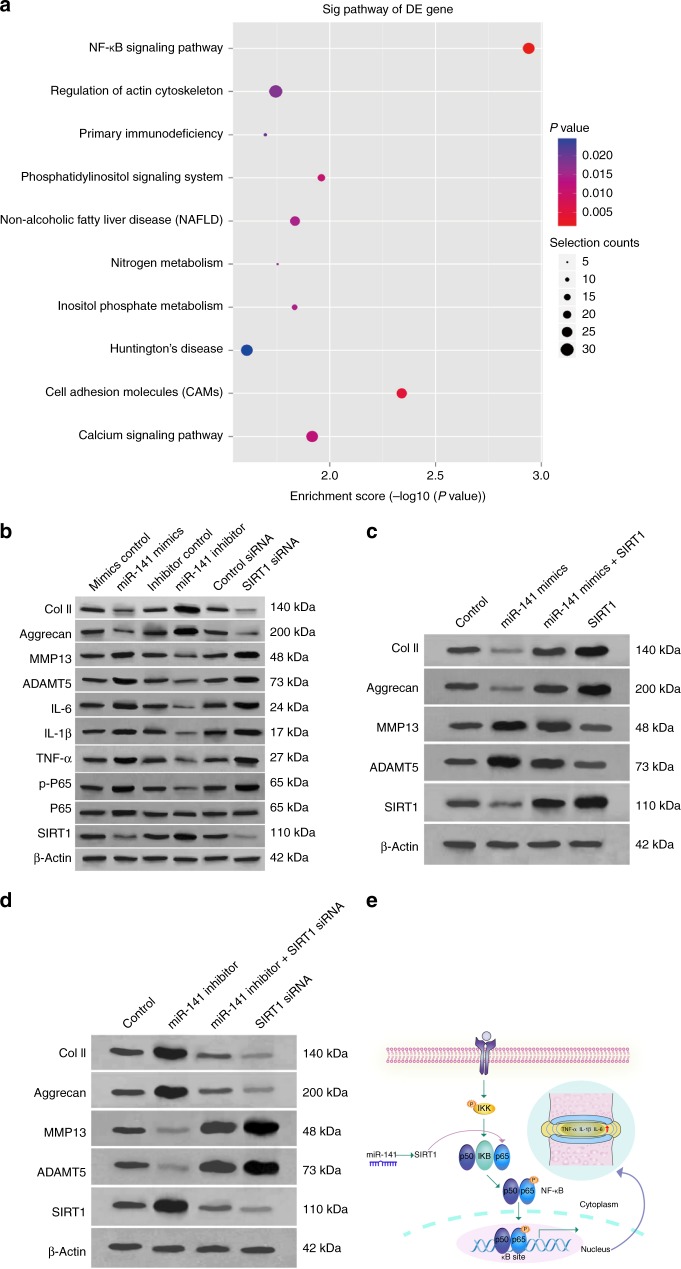
The modulation of miR-141 on SIRT1/NF-κB signaling pathway. **a** KEGG analysis demonstrating NF-κB pathway enriched in IDD. **b** Cultured primary human NP cells were transfected with miR-141 mimics, miR-141 inhibitor, their negative control, control siRNA, or SIRT1 siRNA for 72 h and then the levels of SIRT1, P65, p-P65, TNF-α, IL-1β, IL-6, Col II, aggrecan, MMP13, and ADAMTS-5 were measured by western blotting. **c** The rescue experiments was established in cultured primary human NP cells to validate the relationship between miR-141 and SIRT1. Inhibition of Col II and Aggrecan expression levels by miR-141 mimics was rescued by restoration of SIRT1 expression. In comparison, inhibition of MMP13 and ADAMT5 expression levels by SIRT1 overexpression was rescued by miR-141 mimics. **d** Upregulation of Col II and Aggrecan expression levels by miR-141 inhibitor was abolished by silencing of SIRT1 expression. In comparison, upregulation of MMP13 and ADAMT5 expression levels by silencing of SIRT1 was abolished by miR-141 inhibitor. **e** Schematic representation of mechanisms by which miR-141mediates IDD development. On the basis of findings described in the manuscript, miR-141 downregulates SIRT1 level in NP cells, leading to increased P65 and p-P65. This transcription factor, in turn, leads to increased levels of multiple pro-inflammatory cytokines (TNF-α, IL-1β, IL-6), decreased Col II and aggrecan levels, and increased levels of MMP13 and ADAMTS-5, which induces an imbalance between anabolic and catabolic activities of NP cells. These adverse factors initiate or accelerate IDD

As shown in Fig. 5a, NF-κB signaling pathway was significantly enriched in Kyoto Encyclopedia of Genes and Genomes pathways. Moreover, SIRT1 could directly deacetylate the p65 at lysine 310 residue, thus inhibiting the transactivation capacity of p65 and suppressing NF-κB-dependent gene transcription^16,17^. The findings that miR-141 promotes IDD mediated by SIRT1 prompted us to investigate the potential association between miR-141 and SIRT1/NF-κB pathway. Cultured primary human NP cells were transfected with miR-141 mimics, miR-141 inhibitor, or their negative control, respectively. Expression levels of p-P65, TNF-α, IL-1β, IL-6, MMP13, and ADAMTS-5 were significantly increased in NP cells that stably overexpress miR-141 (Fig. 5b). In contrast, expression levels of p-P65, TNF-α, IL-1β, IL-6, MMP13, and ADAMTS-5 were downregulated in NP cells transfected with miR-141 inhibitor (Fig. 5b). Furthermore, SIRT1 small interfering RNA (siRNA) had effects on p-P65, TNF-α, IL-1β, and IL-6 genes similar to the effects induced by miR-141 (Fig. 5b), indicating that miR-141 regulates IDD progression by targeting the SIRT1/NF-κB pathway. Further experiments were performed to validate the relationship between miR-141 and SIRT1/NF-κB (Fig. 5c, d). These results indicate that miR-141-mediated protection in IDD is primarily through the SIRT1/NF-κB pathway (Fig. 5e).

### Therapeutic use of miR-141 NPs in a mouse model of IDD

**Fig. 6:**
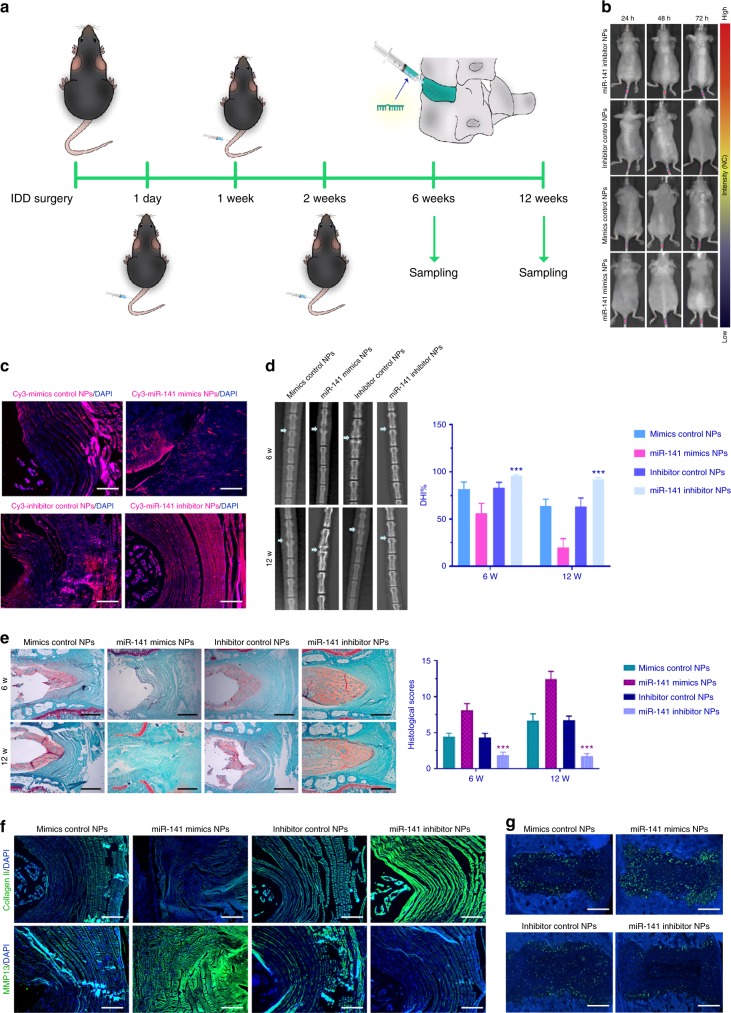
Local delivery of miR-141 inhibitor NPs attenuated IDD development. **a** Overview of the experimental set-up with injections of miR-141 mimics, miR-141 inhibitor, or their negative control NPs at 1, 7, and 14 days after surgery. **b** In vivo time-dependent fluorescence image in mice at 24, 48, and 72 h after the administration of Cy3-miR-141 NPs. The color bar (from blue to red) indicates the change in fluorescence signal intensity from low to high. **c** Cy3-tagged miR-141 NPs analysis. Scale bar = 100 μm. **d**, **e** The intervertebral disc degeneration evaluated by X-ray and Safranin O staining. A significant increase in DHI% was noted at 6 and 12 weeks post-surgery in mice treated by miR-141 inhibitor NPs. Histological score showed a significant decrease in mice treated by miR-141 inhibitor NPs (6 and 12 weeks post-surgery). Scale bar = 50 μm. ***p < 0.001 by one-way ANOVA test followed by Tukey’s post hoc. *n* = 12 per group. **f** Immunostaining for Col II and MMP13 in IDD model treated by miR-141 NPs at 12 weeks. Scale bar = 100 μm. **g** Apoptotic activity determined by TUNEL staining of discs at 12 weeks. Scale bar = 100 μm. Data shown as mean and error bar represents s.e.m.

We sought to investigate the therapeutic role of miR-141 in IDD and to elucidate the underlying molecular mechanisms involved, IDD model was induced in WT mice, followed by local injection of miR-141 mimics or inhibitor NPs and control NPs at 1, 7, and 14 days after surgery (Fig. 6a). The in vivo disc-targeted ability of the NPs was monitored in real time. The delivery of miR-141 mediated by NPs showed an ideal delivery effect (Fig. 6b). Furthermore, Cy3-labeled miR-141 NP analysis showed that miR-141 could penetrate discs (Fig. 6c). Local delivery of miR-141 inhibitor NPs remarkably protected the structure of IVDs as determined by radiographic (Fig. 6d) and histological assessments (Fig. 6e), indicating that silencing of miR-141 had a protective effect against surgically induced IDD. Conversely, mice treated with miR-141 mimics NPs developed severe disc degeneration. Moreover, the expression of MMP13 was significantly decreased by miR-141 inhibitor NPs, whereas an increase in collagen II expression was noted (Fig. 6f). The opposite results were observed in mice treated with miR-141 mimics NPs (Fig. 6f). Terminal deoxynucleotidyl transferase dUTP nick end labeling (TUNEL) staining showed remarkably decreased NP cell apoptosis in mice treated with miR-141 inhibitor NPs (Fig. 6g). Taken together, these results suggest therapeutic effects of silencing of miR-141 on protecting discs from destruction highlighting miR-141 as a potential therapeutic target for IDD.

## Discussion

miR-141, a member of the miR-200 family, has been associated with a variety of human diseases^18–22^. In order to enhance the reliability of results of our article, we set a more stricter selection standard, i.e., miRNAs with more than five-fold upregulation or downregulation were selected for further investigation, which may explain that some miRNAs reported in previous studies (usually two-fold upregulation or downregulation) were not included in our study. A key aspect of our study is that we first provide comprehensive functional and mechanistic characterization of miR-141 in IDD. Disc destruction induced by aging was attenuated by miR-141 KO. In comparison, aging-associated IDD was exacerbated in WT mice, strongly suggesting that miR-141 is an essential regulator of disc degeneration. We then generated an IDD model in miR-141 KO and WT mice. Of note, WT mice were predisposed to the development of IDD with significantly enhanced disc destruction, while mice with miR-141 KO showed protection from disc degeneration, clearly implying that increased miR-141 expression may be a critical factor leading to disc damage in response to pathological conditions. Overall, these findings provide new insights into unrecognized pathophysiological mechanisms underlying disc degeneration.

Needle puncture has been one of the most common methods to establish animal models of IVD degeneration, and disc changes were modulated based on size of the needle relative to the disc height^23,24^. Puncture injury caused by 18, 21, or 23 G needles (needle diameter-to-disc height ratio >0.4) would cause severe damage and degeneration to caudal discs. In comparison, smaller needles (29, 31, 32, or 33 G; needle diameter-to-disc height ratio <0.25) are enough to induce a significant disc degeneration with no effect based on mechanics, biochemical content, disc height, and/or qualitative assays, such as histology. In order to minimize the effect of the procedure itself on discs, 31-G needle was used in our study. With the small ratio of needle diameter to disc height, only neutral zone mechanics were affected in cadaver studies, while no changes were observed in histology, likely due only to partial depressurization of the NP^25–29^. This is further supported by a study where the removal of part or all of the NP, while keeping the AF intact, altered the neutral zone mechanics^30^. Based on the above-mentioned evidence, inflammatory reaction caused by smaller needle may be minor or none. We therefore investigated the inflammatory profile caused by different diameters of needle in mice and found that no acute inflammation (IL-1β, TNF-α, and IL-6) was detected in NP tissues from the 29, 31, and 32 G needle groups, which could exclude the possibility that the procedure itself can produce an inflammatory reaction that may also promote initiation of the degeneration and upregulation of multiple miRNAs.

Although miRNA studies in IDD have been conducted over the years, they have so far not moved into clinical development. One of the biggest challenges in developing miRNA-based therapeutics is to identify the best miRNA candidates or miRNA targets for each disease type. Other challenges include miRNA delivery vehicles that confer higher stability to the therapeutic candidate and enable tissue-specific targeting^13,14,31^. In our study, miR-141 was identified in NP tissues from IDD patients using microarray, followed by two-phase independent cohort validation and FISH analysis. Moreover, using large-scale patient data sets, we demonstrated that miR-141 level is correlated with disc degeneration. More importantly, miR-141 NPs were applied as a highly effective IDD treatment modality. These findings indicate the potential for NPs carrying miR-141 as a promising therapeutic tool for treating IDD.

Accumulating evidence indicates that SIRT1 has an important role in IDD and osteoarthritis (OA) pathogenesis^32–40^. More recently, the pivotal role of SIRT1/NF-κB pathway in age-related diseases including IVD and bone has been recognized^41–47^. Intriguingly, miR-141 interacts with the SIRT1/NF-κB pathway, which implies that the accelerated IDD development in miR-141 overexpression may stem from the activated NF-κB pathway. We verified this point in miR-141 KO mice, suggesting that miR-141 could play a critical role in maintaining homeostasis of discs through associating with this signaling pathway. Furthermore, a dynamic change was found in miR-141 and SIRT1 expression levels in WT mice, indicating that they play important roles in different stages of IDD. The identification of miR-141 as a common upstream regulator of SIRT1/NF-κB pathway is thus of high importance given that, to date, mechanisms of this pathway regulation are still largely unknown. In this report, we identify a clinically relevant, functional miR-141/SIRT1/NF-κB pathway that may be responsible for some of the excessive inflammatory response observed in discs in IDD patients. Because inflammation is present early in the course of IDD, before structural changes have occurred, therapeutic strategies targeting the inflammatory processes might be able to not only halt the progression of IDD but also prevent the onset of radiographic IDD^48–50^. Using the valuable information identified in this study, we may repurpose existing drugs or candidate therapeutics that target this pathway or develop next-generation therapeutics. Before being translated to humans, much work remains to be done and several hurdles need to be overcome. Understanding the side effects and potential off-target effects of vehicles for targeted delivery of miRNAs to specific cell subsets is of key importance. Furthermore, extensive preclinical studies are required to determine the optimal level of inhibition for a given miRNA target, due to the variation in miRNA expression levels across different cell and tissue types under normal physiological conditions as well as in disease. In recent years, our research team have focused on the role of miRNAs in the pathogenesis of degenerative skeletal disorders, including IDD and OA, hopefully establishing the basis for a deeper understanding and an innovative treatment for these diseases. Our previous studies have shown that dysregulated miR-98 and miR-193a-3p were involved in IDD^10,51^. In addition, our recent study demonstrated that inhibition of endogenous miR-218-5p expression/activity appears to be an attractive approach to OA treatment^52^.

Our study highlights the potential clinical application of using miR-141-based therapy to treat IDD patients. Our proof-of-concept studies demonstrate that the NPs carrying miR-141 markedly inhibit disc degeneration progression in IDD model. It would be important, in the future, to further test the combination of NPs carrying miR-141 (or small molecule compounds that can inhibit endogenous expression of miR-141) with the emerging small molecule inhibitors against SIRT1/NF-κB signaling to achieve optimal anti-IDD effect with minimal side effects. Overall, our results indicate that miR-141-based NPs holds great therapeutic potential to treat IDD.

## Methods

### Patient samples

A total of NP samples were obtained from 208 patients (59.6 ± 5.2 years) with degenerative disc disease undergoing discectomy. The surgical indications were as follows: (1) failed conservative treatment and (2) progressive neurologic deficits, such as progressive motor weakness or cauda equine syndrome. Patients with isthmic or degenerative spondylolisthesis, lumbar stenosis, ankylosing spondylitis, or diffuse idiopathic skeletal hyperostosis were excluded. The age- and sex-matched control samples were taken from 163 patients with fresh traumatic lumbar fracture who underwent anterior decompressive surgery due to neurological deficits. Routine magnetic resonance imaging scans of the lumbar spine were taken of these patients before surgery. According to Pfirrmann classification^53^, degree of disc degeneration was graded from T2-weighted images. This study protocol was approved by the ethics committee of affiliated Zhongda Hospital of Southeast University, and written informed consent was obtained from each participant. All ethical guidelines were followed to our description of the experiments performed with human samples.

### Animals

MiR-141 heterozygous KOs on a C57BL/6 background were obtained from the Jackson Laboratory (Bar Harbor, ME, USA). The miR-141 heterozygotes were intercrossed with each other. The miR-141 homozygous KOs WT × WT. The WT and miR-141 KO offspring were used in spontaneous and surgically induced IDD models. For spontaneous IDD experiments, discs were harvested from 6-, 14-, 18-, and 22-month-old miR-141 KO and WT mice. All mice were maintained under pathogen-free conditions.

### Surgically induced IDD model

The IDD model was established in WT C57BL/6 and miR-141 KO mice (12-week-old) by the AF needle puncture^10,33^. A mouse model of tail disc degeneration was chosen as its tail discs have been confirmed to be one of the most similar to human lumbar discs with regarding to disc height, the anteroposterior width, the NP, disc torsion mechanics, axial compression mechanics, and glycosaminoglycan content^54–56^. Furthermore, the morphologic evidence of needle-related mouse tail IVD degeneration reported in several studies is similar to many human age-related DDD features, including radial and concentric anular tears, trabecular bone formation in the endplate, and NP cell changes^33,57,58^. In brief, general anesthesia was administered using ketamine (100 mg/kg). A sagittal small skin incision was performed from Co6 to Co8 to help locate the disc position for needle insertion in the tail. Subsequently, Co6–Co7 coccygeal discs were punctured using a syringe needle. The syringe needle was inserted into Co6–Co7 disc along vertical direction and then rotated in the axial direction by 180° and held for 10 s. The puncture was made parallel to the endplates through the AF into the NP using a 31-G needle, which was inserted 1.5 mm into the disc to depressurize the nucleus. The other segments were left undisturbed as a contrast segment. For spontaneous IDD experiments, discs were harvested at 6 and 12 weeks post surgery from miR-141 KO mice. For the therapeutic experiment, discs were harvested at 6 and 12 weeks post surgery from WT mice. In addition, to determine the acute effect of needle diameter on discs, mice were subjected to 18, 21, 23, 28, 29, and 32 G needle puncture. IL-1β, TNF-α, and IL-6 expression levels were detected in NP tissues 1 and 3 days after needle puncture. All procedures and protocols were approved by the ethics committee of southeast university.

### MiR-141 NP generation and injection

For NP generation, miR-141 (mirVana miRNA mimics/inhibitor) or miRNA negative control (mirVana^TM^ miRNA mimics/inhibitor Negative Control #1, catalog number: 4464061/4464079) (Life Technologies) labeled with Cy3 using the Silencer® siRNA Labeling Kit (#AM1636) was packed in a NP carrier system (MaxSuppressor In Vivo RNALANCEr II Kit and Lipid Extruder, BIOO Scientific) according to the manufacturer's instructions. Based on this, Cy3-mimics control NPs, Cy3-miR-141 mimics NPs, Cy3-inhibitor control NPs, or Cy3-miR-141 inhibitor NPs was obtained.

For the therapeutic experiment, a total of 48 male mice (12-week-old C57BL/6) (Jackson Laboratory, Bar Harbor, USA) undergoing IDD surgery were assigned to four groups (*n* = 12 per group), i.e., Cy3-mimics control, Cy3-miR-141 mimics, Cy3-inhibitor control, or Cy3-miR-141 inhibitor NP-treating groups. In Cy3-mimics control NP-treating group or Cy3-miR-141 mimics NP-treating group, mice were injected locally with 20 μL Cy3-mimics control NPs or 20 μL Cy3-miR-141 mimics NPs on days 1, 7, and 14 after IDD surgery. In Cy3-inhibitor control NP-treating group or Cy3-miR-141 inhibitor NP-treating group, mice were treated with the local delivery of 20 μL Cy3-inhibitor control NPs or 20 μL Cy3-miR-141 inhibitor NPs on days 1, 7, and 14 after IDD surgery. To determine the transfection efficiency of miR-141 mimics/inhibitor or its negative control labeled with Cy3, in vivo fluorescence imaging using an IVIS 200 Imaging system (Xenogen, Caliper Life Science, MA, USA) and histology were performed at different time points postinjection (24, 48, and 72 h) in each group. At 6 and 12 weeks after surgery, discs were harvested for histological and radiographic evaluation in each group.

### Histological and radiographic evaluation

The discs from mice were fixed in 10% neutral-buffered formalin for 1 week, decalcified in EDTA for 2 weeks, paraffin-embedded, and carefully sectioned to a 5-μm thickness. Midsagittal sections were stained with hematoxylin and eosin and Safranin O-fast green. The histological images were analyzed using the Olympus BX51 microscope (Olympus Center Valley, PA, USA). Based on a literature review of disc degeneration studies^58–64^, a modified histologic grading system was developed. More specifically, the cellularity and morphology of the AF, NP, and the border between the two structures were examined. The scale is based on 5 categories of degenerative changes with scores ranging from 0 points (0 in each category) for a normal disc to 15 points (3 in each category) for a severely degenerated disc. For morphology of the NP, score 0: round shape and the NP constitutes >75% of the disc area, score 1: round shape and the NP constitutes 50–75% of the disc area, score 2: round shape and the NP constitutes 25–50% of the disc area, score 3: round shape and the NP constitutes <25% of the disc area. For cellularity of the NP, score 0: stellar-shaped cells with a proteoglycan matrix located at the periphery, evenly distributed, score 1: partially stellar and partially round cells, more stellar than round, score 2: mostly large, round cells, separated by dense areas of proteoglycan matrix, score 3: large, round cells, separated by dense areas of proteoglycan matrix. For morphology of the AF, score 0: well-organized collagen lamellae with no ruptures, score 1: inward bulging, ruptured, or serpentine fibers constitute <25% of the AF, score 2: inward bulging, ruptured, or serpentine fibers constitute 25−50% of the AF, score 3: inward bulging, ruptured, or serpentine fibers constitute >50% of the AF. For cellularity of the AF, score 0: fibroblasts comprise >90% of the cells, score 1: fibroblasts comprise >75–90% of the cells, score 2: intermediate, score 3: chondrocytes comprise >75% of the cells. For border between the NP and AF, score 0: normal, without any interruption, score 1: minimal interruption, score 2: moderate interruption, score 3: severe interruption. Radiographs were taken at 6 and 12 weeks after the puncture. The change in IVD height was evaluated by the disc height index (DHI)^58,60^. Measurements of internal control discs were carried out together with their corresponding punctured discs. Disc height and the adjacent vertebral body heights were measured on the midline and 25% of the disc’s width from the midline on either side. The DHI was expressed as the mean of the 3 measurements from midline to the boundary of the central 50% of disc width divided by the mean of the 2 adjacent vertebral body heights. Changes in the DHI of punctured discs were expressed as a percentage (%DHI = post-punctured DHI/pre-punctured DHI × 100).

### Isolation and three-dimensional culture of NP cells

The human disc tissue specimens were first washed twice with phosphate-buffered saline (PBS), and NP was separated from the AF using a stereotaxic microscope, cut into pieces (2–3 mm^3^), and the NP cells were released from the NP tissues by incubation with 0.25 mg/mL type II collagenase (Invitrogen, Carlsbad, CA, USA) for 12 h at 37 °C in Dulbecco’s modified Eagle medium (DMEM; GIBCO, Grand Island, NY, USA). Matrigel (BD Biosciences, San Jose, CA) was thawed overnight at 4 °C, coated the Costar Transwell inserts (Corning, Corning, NY) with 60 μL each, and the gel was allowed to solidify and dry at 37 °C for 2 h. NP cells were seeded at 0.5 × 10^6^ cells per Transwell arranged in 24-well plate and cultured in F12 basal medium supplemented with 2.5% matrigel, 10% fetal bovine serum plus 2.5 mg/mL L-ascorbic acid-2-phosphate (sigma). Medium was changed twice per week for the duration of cell pellet culture.

### RNA isolation, cDNA synthesis, and qRT-PCR

Total RNA including miRNA was isolated with TRIzol Reagent (Ambion, Life Technologies, Carlsbad, CA, USA) from tissues or cultured cells according to the manufacturer’s instructions. RNA quantity and quality were determined using a nanodrop (Thermo Scientific, Waltham, MA, USA) and Bioanalyzer (Agilent Inc., Santa Clara, CA, USA). For quantitative detection of miRNA, TaqMan MiRNA assays (Applied Biosystems), iScript Select cDNA Synthesis Kit (Bio-Rad), and iQSupermix (Bio-Rad) Kits were used according to the manufacturer’s instructions. U6 snRNA was used as control for normalization. For quantitative detection of mRNA levels, complementary DNAs were synthesized by using oligo-dT primers and the iScript Select cDNA Synthesis Kit (Bio-Rad). Real-time PCR analyses were performed using specific set of primers and iQSYBR Green mix (Bio-Rad). β-Actin level was used for normalization of the gene-specific expression levels. All reactions were run on a real-time PCR system (Applied Biosystems) and analyzed using the comparative Ct (ΔΔCt) method (2^−ΔΔCt^ with logarithm transformation). The specific primers are listed in Supplementary Tables 2 and 3.

### Microarray analyses

Total RNAs (50 ng) were labeled and amplified using the Low Input Quick Amp Labeling Kit (Agilent Technologies). The Cy3-labeled RNAs were resuspended in 40 μL of hybridization solution (Agilent Technologies), applied to a SurePrint G3 Human GE 8 × 60 K array (Agilent Technologies), and covered with a Gasket 8-plex slide (Agilent Technologies). The slides were hybridized for 17 h at 65 °C, washed with Gene Expression Wash Buffer 1 (Agilent Technologies) for 1 min at room temperature (RT), washed with Gene Expression Wash Buffer 2 (Agilent Technologies) for 1 min at 37 °C, and then air-dried. Agilent Feature Extraction software (version 11.0.1.1) was used to analyze acquired array images. Quantile normalization and subsequent data processing were performed by using the GeneSpring GX v12.1 software package (Agilent Technologies). After quantile normalization of the raw data, miRNAs that at least three out of the six samples have flags in Detected (All Targets Value) were chosen for further data analysis. Differentially expressed miRNAs with statistical significance between the two groups were identified through Volcano Plot filtering. Differentially expressed miRNAs between the two samples were identified through Fold Change filtering. Hierarchical cluster analysis was conducted using the Gene Cluster 3.0 software (Stanford University). Functional group analysis was performed using Database for Annotation, Visualization, and Integrated Discovery (DAVID 6.7). The *p* value was set to 0.05 to denote the significance of GO enrichment in the differentially expressed mRNA list. Fold enrichment ([Count/Pop.Hits/List.Total/Pop.Total]) was employed to denote the enrichment of a particular GO term.

### In vitro miRNA and siRNA transfection

The cultured primary human NP cells were transfected with mimics or inhibitor labeled or unlabeled with Cy3 using the Silencer® siRNA Labeling Kit (#AM1636) or miRNA negative control (mirVanaTM miRNA mimics/inhibitor Negative Control #1, catalog number: 4464061/4464079) (Life Technologies) at 50 nM using Lipofectamine RNAiMAX Transfection Reagent (Invitrogen). To suppress SIRT1 expression, cells were transfected with either SIRT1 siRNA or control scrambled siRNA (Thermo Scientific Dharmacon®) using Lipofectamine 3000 (Invitrogen) according to the manufacturer's instructions. The SIRT1 expression plasmid (pcDNA™3.1/V5-His TOPO™ TA Expression Kit) was obtained (Invitrogen™). At 48 h after transfection, the cellular lysates were collected to analyze the expression of genes of interest.

### 3′-Untranslated region (UTR) cloning and luciferase assay

To construct the WT SIRT1 3’UTR-Luc reporter plasmid (SIRT1 3′UTR), a fragment of the 3′UTR of the SIRT1 gene, including the predicted miR-141-binding site, was PCR-amplified and then cloned into the psi-CHECKTM-2 vector (Promega, Madison, WI) downstream of the firefly luciferase gene with XhoI and NotI (Thermo Fisher Scientific). To produce constructs that bear mutations at a putative miR-141-binding site in WT SIRT1 3′UTR, site-directed mutagenesis was performed using the QuikChange Lightning Site-Directed Mutagenesis Kit (Agilent Technologies, Inc., Santa Clara, CA, USA). The PCR mixture had 0.7 μL of expand long-range enzyme mix (Roche, Mannheim, Germany), 10 μL of 5× expand long-range buffer, 100 ng of plasmid template, 100 nM of primers, 3 μL of dimethyl sulfoxide, and 2.5 μL of dNTPs (10 mM). PCR cycling conditions were as follows: 92 °C for 30 s, 55 °C for 1 min, 68 °C for 10 min, and a final extension at 68 °C for 10 min. After PCR, 20 μL of the reaction was digested with DpnI at 37 °C for 1 h and 10 μL was transformed into DH5 alpha *Escherichia coli* to prepare the mutant construct plasmids. All constructs were confirmed by sequencing (Cosmogenetech, Seoul, Korea). For luciferase assay, cultured primary human NP cells were seeded at 3000 cells per well in a 96-well plate. Cells were co-transfected with WT- or mutant-type SIRT1 3′UTR-Luc reporter plasmid and miR-control or miR-141 using Lipofectamine PLUS^TM^ reagent (Invitrogen). Cell lysates were harvested 48 h after transfection and luciferase activity was assayed with the Dual-Glo Luciferase Assay system (Promega, Madison, WI, USA) according to the manufacturer’s instructions. Luciferase activity was normalized by firefly luciferase activity. Experiments were performed in triplicate and repeated at least three times independently.

### Flow cytometry (FCM)

Apoptosis was evaluated by staining cultured primary human NP cells with both Annexin V-fluorescein isothiocyanate (FITC) and propidium iodide (PI), according to the manufacturer’s instructions. Annexin V-FITC was employed to quantitatively determine the percentage of cells undergoing apoptosis. It relies on the property of cells to lose membrane asymmetry in the early phase of apoptosis. In apoptotic cells, the membrane phospholipid phosphatidylserine is translocated from the inner leaflet of the plasma membrane to the outer leaflet, thereby exposing phosphatidylserine to the external environment. Cells that were positively stained with Annexin V-FITC and negatively stained for PI were considered apoptotic. Cells that were positively stained for both Annexin V-FITC and PI were considered necrotic. The cells were stained with 5 µL Annexin V-FITC and 10 µL PI and then analyzed with EpicsAltra (Beckman Coulter, CA, USA) FCM.

### Cell Counting Kit-8 (CCK8) and EdU assay

Cell proliferation was measured using the CCK8 (Dojindo Molecular Technologies, Inc., Kumamoto, Japan) following the manufacturer’s instructions. The cultured primary human NP cells were seeded in 96-well plates at the density of 1000 cells per well with 100 mL of complete culture medium and transfected with miR-control, miR-141 mimics, or miR-141 inhibitor. The cells were then cultured for another 24, 48, and 72 h. The supernatant was removed, and 100 mL of DMEM/F12 medium containing 10 mL of CCK8 was added to each well for incubation for another 3 h at 37 °C. The culture plates were then shaken for 10 min and the optical density values were read at 450 nm. Each sample was analyzed in triplicate. For EdU assay, cultured primary human NP cells were inoculated at a density of 2 × 10^5^ per well into 24-well plates and cultured at 37 °C in 5% CO_2_. In all, 50 μM of EdU (Sigma-Aldrich) was then added to each well for 2 h. Next, cells were fixed with 4% formaldehyde for 15 min, followed by permeabilization with 0.5% Triton X-100 for 20 min at RT. After washing the cells three times with PBS, 100 μL of 1× Apollo reaction cocktail was added to each well for 30 min at RT. Subsequently, the cells were stained with Hoechst 33258. The EdU incorporation rate was expressed as the ratio of EdU-positive cells (red cells) to total Hoechst 33258-positive cells (blue cells).

### Fluorescence in situ hybridization

A locked nucleic acid (LNA) probe with complementarity to miR-141 was labeled with 5′ and 3′-digoxigenin and synthesized by Exiqon (Woburn, MA, USA). A scrambled LNA probe was used as a negative control. The NP tissues from IDD patients were used for FISH detection. The slides were prehybridized for 30 min at 52 °C and then 10 pmol of the probe in hybridization mixture was added to each slide and incubated for 1 h at 52 °C. Slides were incubated with 3% (vol/vol) H_2_O_2_ for 10 min at RT to block endogenous peroxidases before applying horseradish peroxidase (HRP)-conjugated antibodies. After washing, slides were incubated in blocking buffer for 30 min at RT, then antibody was added, and slides were incubated for 30 min at RT. The TSA Plus Fluorescein System (PerkinElmer, Waltham, Massachusetts, USA) was used for direct fluorescence detection according to the manufacturer’s protocol. The slides were imaged using an epifluorescence microscope equipped with charge-coupled device camera and image analysis capabilities. Tissue sections were mounted in Vectashield (Vector laboratories). Images were taken with FV1000 confocal laser-scanning microscope (Olympus IX-81; Olympus, Tokyo, Japan). The intensities of miR-141 staining was scored by 0–4^65^, according to the standards of no staining = 0, minimal staining = 1 + , weak staining = 2+, moderate staining = 3+, or strong staining = 4+. The percentages of miR-141 cells in three representative high-power fields of individual samples were analyzed. Those expression scores equaled to scores of the intensities × the percentages of positive cells, and the maximum was 4 and the minimum was 0. Every samples were assessed by at least two pathologists in a blinded manner, and those expression scores of ≥2 was defined as high expression, <2 was low expression.

### Western blotting

Protein lysates were prepared from cultured primary human NP cells using RIPA buffer supplemented with protease and phosphatase inhibitors. The protein concentrations were determined using a BCA Protein Assay Reagent Kit (Pierce Biotechnology, Rockford, IL, USA). The lysates were loaded and separated on 10% sodium dodecyl sulfate-polyacrylamide gels. Nitrocellulose membranes were subsequently probed with primary antibodies against anti-Col II antibody (diluted 1:400, Abcam, catalog number: ab34712), anti-Aggrecan antibody (diluted 1:1000, Abcam, catalog number: ab36861), anti-ADAMTS-5 antibody (diluted 1:250, Abcam, catalog number: ab41037), anti-MMP13 antibody (diluted 1:500; Abcam, catalog number: ab39012), anti-SIRT1 antibody (diluted 1:2000; Abcam, catalog number: ab32441), anti-P65 antibody (diluted 1:1500; Abcam, catalog number: ab16502), anti-p-P65 antibody (diluted 1:500; Abcam, catalog number: ab28856), anti-TNF-α antibody (diluted 1:500; Abcam, catalog number: ab6671), anti-IL-1β antibody (diluted 1:1000; Abcam, catalog number: ab200478), anti-IL-6 antibody (diluted 1:2000, Cell Signaling Technology, catalog number: 5216), and anti-beta-actin (diluted 1:2000, Cell Signaling Technology, catalog number: 4967) in 5% bovine serum albumin (BSA) in TBS-T overnight at 4 °C. After washing with TBS-T, the membranes were incubated with HRP-linked anti-rabbit immunoglobulin G (IgG; diluted 1:1000, Cell Signaling Technology, catalog number: 7074) or HRP-linked anti-mouse IgG (diluted 1:1000, Cell Signaling Technology, catalog number: 7076) for 2 h. Protein bands were detected using an EzWestLumi ECL solution (ATTO Corporation, Tokyo, Japan) as per the manufacturer’s specifications (ATTO Corporation, Ez-Capture II). Densities of protein bands were measured using the CS Analyzer software (Version 3.00.1011, ATTO & Rise Corporation). The experiment was performed at least in triplicate. Full scans of important western blots are provided in Supplementary Fig. 6.

### Cell immunofluorescence

The cultured primary human NP cells were fixed in 4% paraformaldehyde, permeabilized with PBS containing 0.25% Triton X-100 for 10 min, and then blocked with 4% BSA containing 0.25% Triton X-100 for 30 min at RT. The cells were incubated with primary antibodies against Col II (diluted 1:2000, Abcam, catalog number: ab34712), Aggrecan (diluted 1:500, Abcam, catalog number: ab36861), MMP13 (diluted 1:200, Abcam, catalog number: ab39012), and ADAMTS-5 (diluted 1:1000, Abcam, catalog number: ab41037) overnight at 4 °C. The cells were rinsed three times with PBS and incubated with goat anti-rabbit IgG (H&L) conjugated with Alexa Fluor 488 (diluted 1:500, Abcam, catalog number: ab150077). After washing, the nuclei were counterstained with 4,6-diamidino-2-phenylindole (DAPI; Invitrogen) for 5 min. The fluorescence was visualized under CarlZeiss LSM710 confocal microscope (CarlZeiss, Oberkochen, Germany). Images were analyzed using the Image-Pro Plus 6.0 software (Media Cybernetics, Silver Spring, MD, USA).

### Immunofluorescent staining of histological sections and TUNEL staining

Frozen sections of mice discs were fixed with 4% paraformaldehyde for 5 min. After washing with TBS-T, the sections were incubated with 10 mg/mL hyaluronidase (Sigma) at 37 °C for 30 min. After blocking with 10% goat normal serum (Nichirei Corporation), the sections were incubated with primary antibody for 2 h at RT. Paraffin-embedded sections were deparaffinized and incubated in 1 mM EDTA (pH 8.0) at 80 °C for 15 min to retrieve the antigen. Then sections were treated with 10 mg/mL hyaluronidase at 37 °C for 30 min. The primary antibodies used were as follows: anti-MMP13 (diluted 1:200, Abcam, catalog number: ab39012), anti-Col II (diluted 1:2000, Abcam, catalog number: ab34712), and anti-SIRT1 (diluted 1:500, Abcam, catalog number: ab157401). Immune complexes were detected using secondary antibodies. DAPI (Invitrogen) was used. To detect NP cell apoptosis, TUNEL staining was performed using a kit based on the manufacturer’s instruction (Promega, Fitchburg, WI, USA).

### Statistical analysis

For statistical analysis, the GraphPad Prism 7 Software (GraphPad Software, San Diego, CA, USA) was used. For the results of qRT-PCR, we employed Mann–Whitney *U* test. Comparison of statistical difference between the two experimental groups was determined by two-tailed unpaired Student’s *t* test. Statistical analysis comparing multiple groups with one-way analysis of variance followed by Tukey’s post hoc. A *p* value of <0.05 was considered statistically significant.

## Electronic supplementary material

Supplementary Information

Reporting Summary

## Data Availability

The microarray data have been deposited in the GEO database under the accession code (GSE116726). We declare that the data supporting the findings of this study are available within the article and its supplementary information files and from the corresponding author upon reasonable request.
