# Supplementary material for: Preclinical development of a microRNA-based therapy for intervertebral disc degeneration
Source: Nat Commun. 2018 Nov 28;9:5051. doi: 10.1038/s41467-018-07360-1 (PMC6262020; doi:10.1038/s41467-018-07360-1)
Supplement: Supplementary file 1 — Supplementary Information [file 41467_2018_7360_MOESM1_ESM.pdf]

Preclinical development of a microRNA-based therapy for intervertebral disc degeneration

Ji et al.

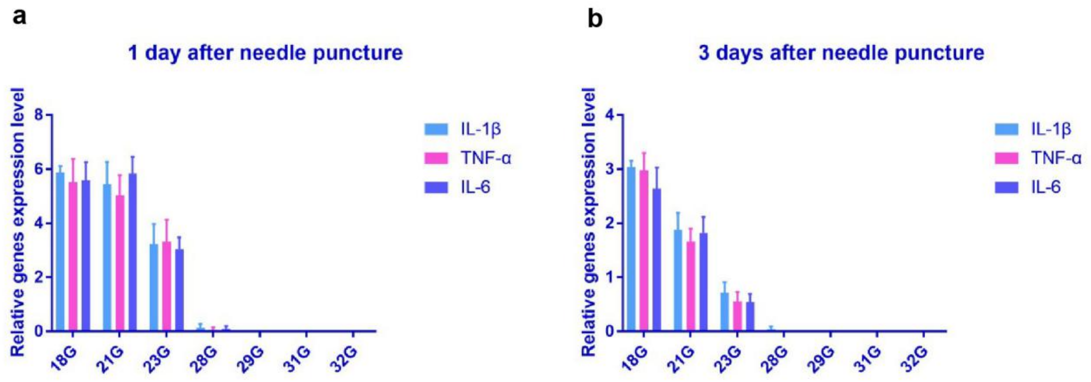

Supplementary Fig. 1 The effect of different diameter of needle on disc profile. (a and b) No acute inflammation (IL-1β, TNF-α and IL-6) was detected in NP tissues from 29G, 31G and 32G needle groups at 1 and 3 days after needle puncture. In comparison, IL-1β, TNF-α and IL-6 expression levels were high in 18G, 21G, 23G needle groups. Low IL-1β, TNF-α and IL-6 expression levels were observed in 28G needle group.

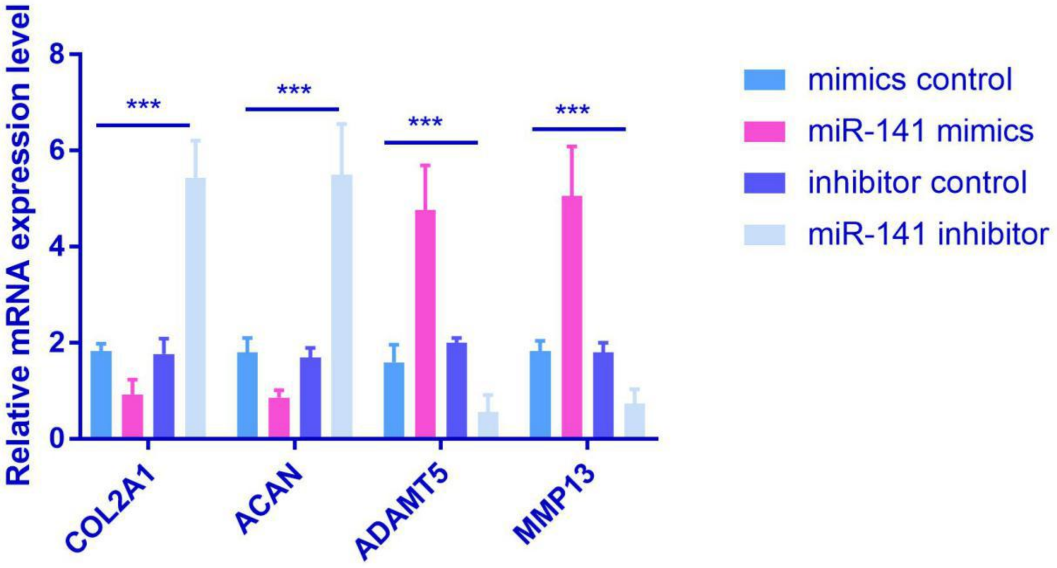

Supplementary Fig. 2 The effect of miR-141 on disc metabolism. Downregulation of miR-141 increased COL2A1 and ACAN expression, whereas upregulation of miR-141 increased MMP13 and ADAMT5. ***$p < 0.001$ by one-way ANOVA test followed by Tukey's post-hoc.

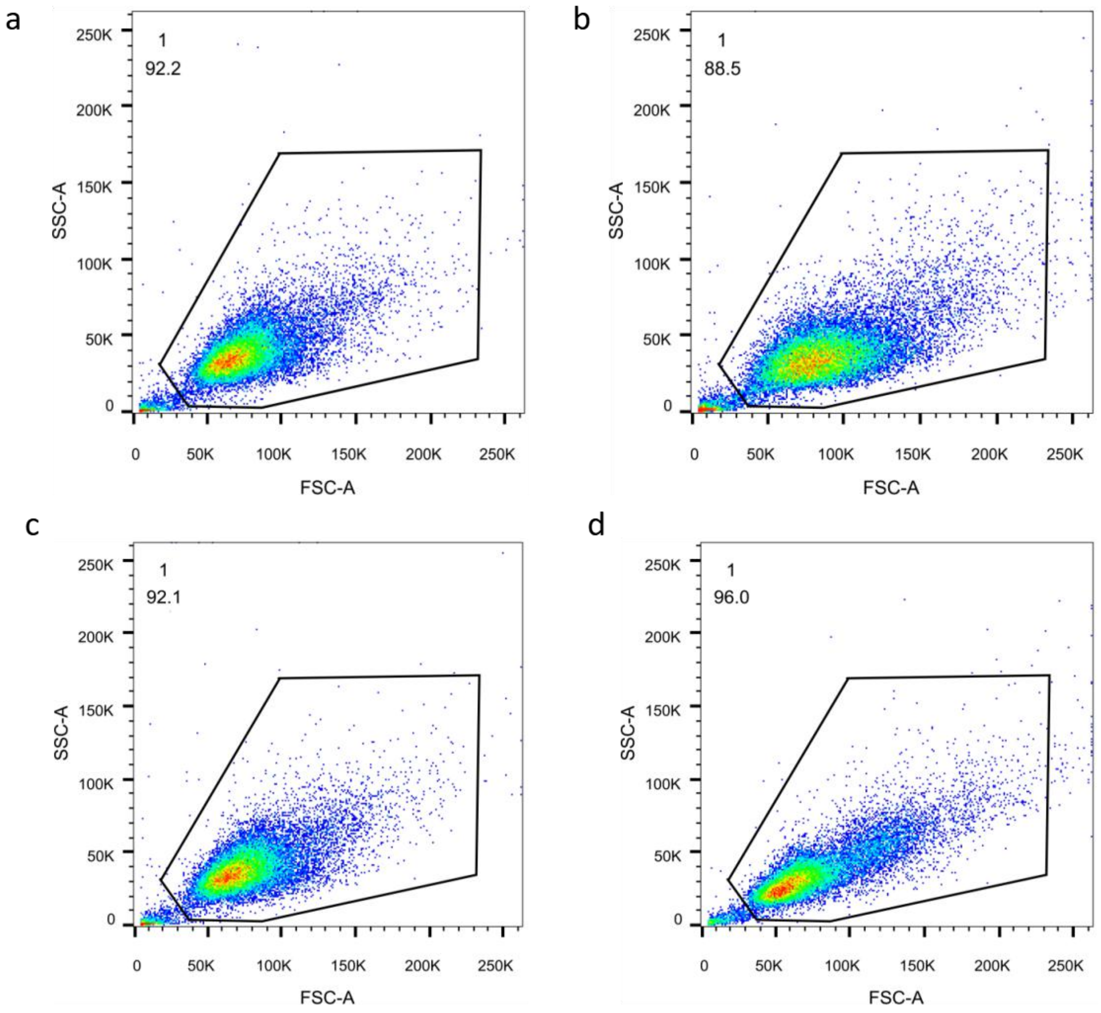

Supplementary Fig. 3 Gating strategies used for cell sorting. (a) Gating strategy to sort apoptotic cells under mimics control treatment. **(b)** Gating strategy to sort apoptotic cells under miR-141 mimics treatment. **(c)** Gating strategy to sort apoptotic cells under inhibitor control treatment. **(d)** Gating strategy to sort apoptotic cells under miR-141 inhibitor treatment.

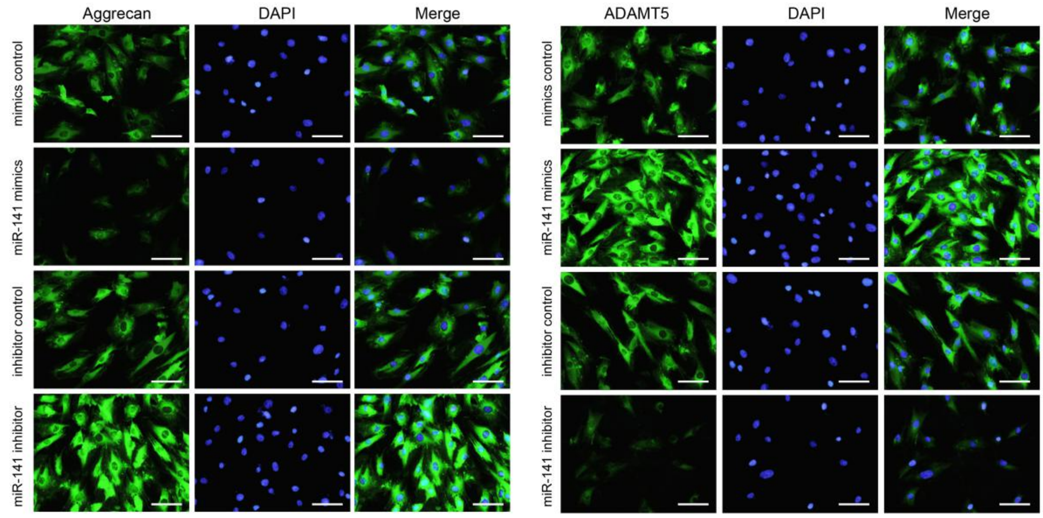

Supplementary Fig. 4 Immunofluorescence for Aggrecan and ADAMT5. Scale bar=100μm.

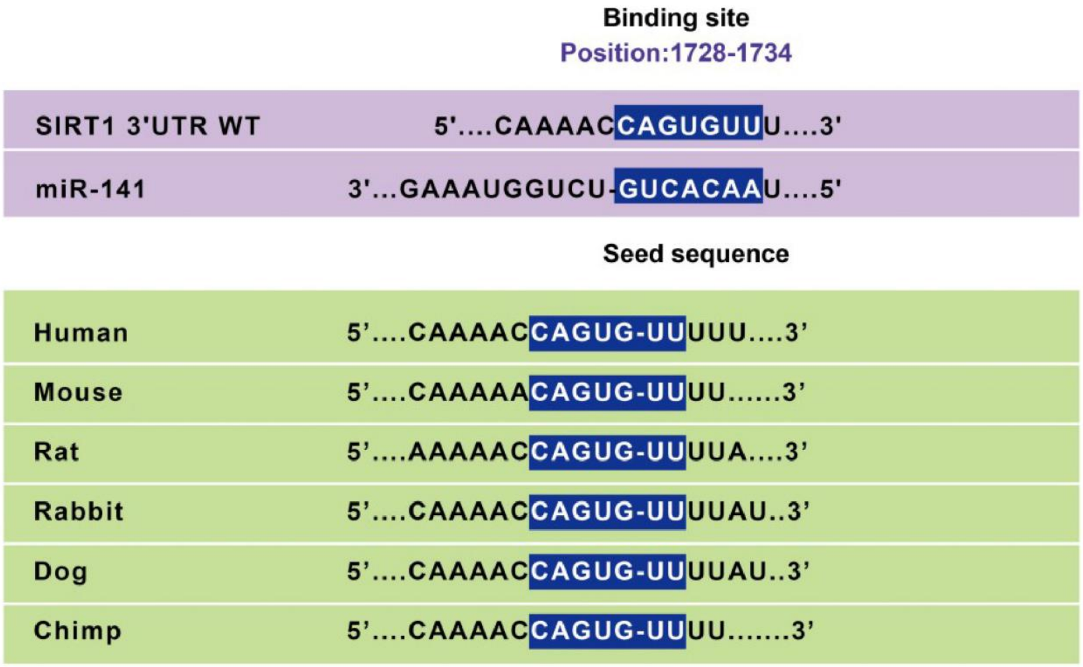

Supplementary Fig. 5 The conserved sequence of miR-141 binding sites in 3'UTR SIRT1 gene shown from several species

Fig. 5b

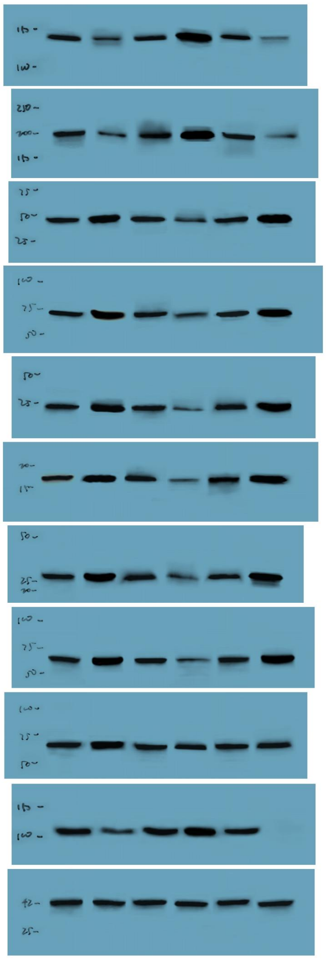

Fig. 3f

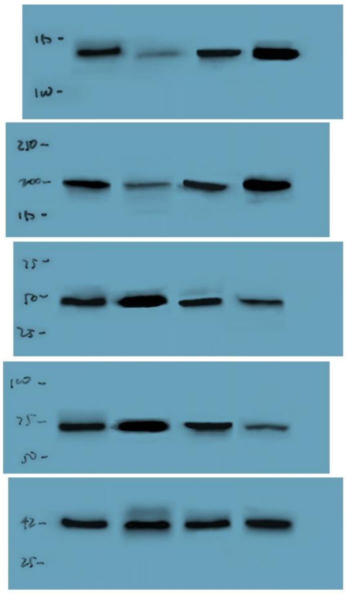

Fig. 4i

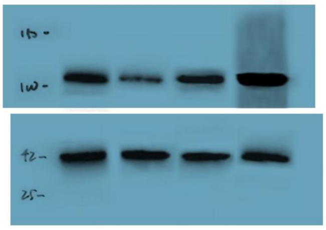

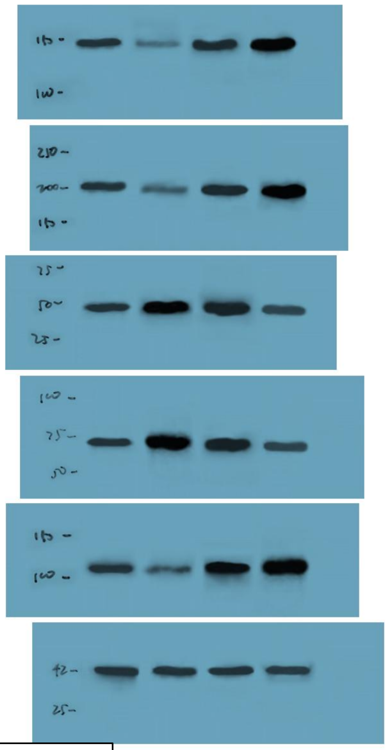

Fig. 5c

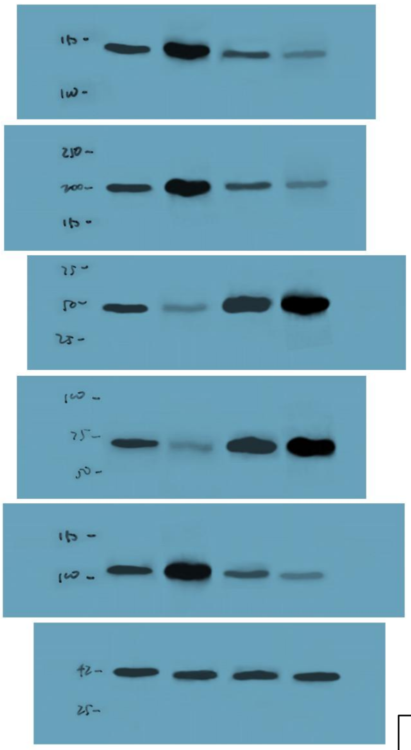

Fig. 5d

Supplementary Fig.6 Full scans of the immunoblots.

Supplementary Table 1 Differentially expressed miRNAs in NP tissues from IDD and controls in both one- and two-stage validation

miRNAs	One stage		Two stage	
	Fold change	P Value	Fold change	P Value
**Up-regulated**				
hsa-miR-563	4.2	0.09	-	-
**hsa-miR-146a**	6.8	0.002**	5.6	0.17
hsa-miR-29c	2.2	0.31	-	-
hsa-miR-34a	4.9	0.06	-	-
hsa-miR-610	7.2	0.47	-	-
hsa-miR-187	5.1	0.04	-	-
hsa-miR-127-5p	6.9	0.78	-	-
hsa-miR-192	7	0.05	-	-
**hsa-miR-141**	**7.6**	**0.008****	**7.3**	**0.002****
hsa-miR-139-5p	3.6	0.25	-	-
hsa-miR-338-3p	2.7	0.07	-	-
hsa-miR-455-5p	4.4	0.61	-	-
**hsa-miR-21**	5.9	0.006**	5.2	0.21
**Down-regulated**				
**hsa-miR-378**	0.12	0.004**	0.16	0.39
hsa-miR-590-5p	0.34	0.16	-	-
hsa-miR-410	0.22	0.37	-	-
hsa-miR-486	0.03	0.06	-	-
hsa-miR-204	0.11	0.28	-	-
hsa-miR-376a-5p	0.27	0.43	-	-
hsa-miR-32	0.33	0.02	-	-
hsa-miR-181c	0.04	0.08	-	-

NP: nucleus pulposus; IDD: intervertebral disc degeneration; Hsa: human; ** P < 0.01 by by Mann-Whitney U test.

Supplementary Table 2 Primers used for quantitative real-time polymerase chain reaction. Primers were designed from the mouse genome.

Gene	Forward Primers	Reverse Primers
miR-141	CATCCGATTAACACTGTCTGGTAA	TATGGTTGTTCTGCTCTCTGTCTC
U6	ATTGGAACGATACAGAGAAGATT	GGAACGCTTCACGAATTTG
SIRT1	GGCTACCGAGGTCCATATACTTTTG	TCAGGTGGAGGAATTGTTTCTGG
IL-1β	GCTGAAAGCTCTCCACCTCAATG	TGTCGTTGCTTGGTTCTCCTTG
TNF-α	TCTACTGAACTTCGGGGTGATCG	AGATGATCTGAGTGTGAGGGTCTGG
IL-6	AACAAGAAAGACAAAGCCAGAGTCC	TCTGTTAGGAGAGCATTGGAAATTG

Supplementary Tables 3 Primers used for quantitative real-time polymerase chain reaction. All primers were designed from the human genome

Gene	Forward Primers	Reverse Primers
miR-141	CATCCGATTAACACTGTCTGGTAA	TATGGTTGTTCTGCTCTCTGTCTC
U6	ATTGGAACGATACAGAGAAGATT	GGAACGCTTCACGAATTTG
SIRT1	TTCCCTCAAAGTAAGACCAGTAGC	TTGGCATATTCACCACCTAACC
COL2A1	TCCCAGAACATCACCTACCAC	CCATCCTTCAGGGCAGTGTA
ACAN	ACAATGCCCAAGACTACCAG	GTGCCAGATCATCACCACA
ADAMT5	AGTTTGCCTATCGTCACTGTAAT	GTAGATGGCCCTCTTCCCT
MMP13	GGAAGACCTCCAGTTTGCAGAGC	GCTGCATTCTCCTTCAGGATTCC
